# Molecular cytogenetic characterization and comparison of the two cultivated *Canavalia* species (Fabaceae)

**DOI:** 10.3897/CompCytogen.v11i4.13604

**Published:** 2017-09-12

**Authors:** Chao-Wen She, Lin Wei, Xiang-Hui Jiang

**Affiliations:** 1 Key Laboratory of Research and Utilization of Ethnomedicinal Plant Resources of Hunan Province, Huaihua University, Huaihua, Hunan, 418008, China; 2 Key Laboratory of Xiangxi Medicinal Plant and Ethnobotany of Hunan Higher Education, Huaihua University, Huaihua, Hunan, 418008, China; 3 College of Biological and Food Engineering, Huaihua University, Huaihua, Hunan, 418008, China

**Keywords:** *Canavalia* cultivars, karyotype, 5S rDNA, 45S rDNA, fluorochrome banding, *in situ* hybridization

## Abstract

The two cultivated *Canavalia* (Adanson, 1763) species, *Canavalia gladiata* (N. J. von Jacquin, 1788) A. P. de Candolle, 1825 and *Canavalia ensiformis* (Linnaeus, 1753) A. P. de Candolle, 1825 are closely related based on morphological and molecular phylogenetic data. However, the similarities and differences in genome organization between them have not been evaluated at molecular cytogenetic level. Here, detailed karyotypes of both species were constructed using combined PI and DAPI (CPD) staining, rDNA-FISH and self-genomic *in situ* hybridization (sGISH). For further comparison, comparative genomic *in situ* hybridization (cGISH) and sequence analysis of 5S rDNA were applied. Their chromosomes were accurately identified by sGISH and rDNA-FISH signals. Both species had the karyotype formula 2n = 22 = 18m + 4m-SAT, but the karyotype of *C. ensiformis* was shorter and more asymmetric than that of *C. gladiata*. They displayed similar CPD bands at all 45S rDNA sites and centromeres. *C. gladiata* had ten centromeric 5S rDNA loci and two SC (secondary constriction)-associated 45S rDNA loci. *C. ensiformis* had nine centromeric and one interstitial 5S loci, two SC-associated and one proximal 45S loci. Their sGISH signal patterns displayed both basic similarities and distinct differences. Reciprocal cGISH generated prominent signals in all pericentromeric regions and 45S sites. There was lower level of sequence identity of the non-transcribed spacer between their 5S rDNA repeats. These data confirmed the evolutionary closeness between *C. gladiata* and *C. ensiformis* and demonstrated obvious differentiation between their genomes, and supported the opinion that *C. ensiformis* is more advanced in evolution than *C. gladiata*.

## Introduction

The genus *Canavalia* Adanson, 1763, belonging to the tribe Diocleae of the family Fabaceae, comprises about sixty pantropical species (Smartt 1990, Snak et al. 2016). This genus has two cultivated species, *Canavalia gladiata* (N. J. von Jacquin, 1788) A. P. de Candolle, 1825 (sword bean) and *Canavalia ensiformis* (Linnaeus, 1753) A. P. de Candolle, 1825 (jack bean). *C. gladiata* was domesticated in Asia and widely cultivated in the tropics whereas *C. ensiformis* is native to Central America and the West Indies and is widely cultivated in tropical and subtropical regions (Smartt 1990). Both are raised as food, forage, green manure, and cover crops to control erosion (Smartt 1990, Ekanayake et al. 2000). Their young seeds and immature pods are cooked and eaten as vegetables. The seeds of *C. gladiata* are used in Chinese herbal medicine as a treatment for cold, hiccups and vomiting (Chinese Pharmacopoeia Commission 2015). The seeds of *C. ensiformis* are a source of concanavalin A (Morris 2007).

Although *C. gladiata* and *C. ensiformis* differ in geographical origin, they are closely related. This fact was established by their highly similar morphologies and seed proteins (Smartt 1990), and the molecular phylogenetic tree (Snak et al. 2016). Purseglove (1974) suggested that *Canavalia virosa* (Roxburgh, 1814) Wight & Arnott, 1833, a wild bean found in tropical Asia and Africa, is the ancestral form of *C. gladiata*. No such progenitor has been suggested for *C. ensiformis* among New World species. Westphal (1974) suggested that *C. gladiata*, *C. ensiformis*, and *C. virosa* are so morphologically similar that in effect they constitute a single species. Therefore, they may, in fact, be geographical or domesticated races within a single biological species (Smartt 1990). Testing these hypotheses at cytogenetic and molecular levels is straightforward. However, there is very little cytogenetic and molecular data available for *Canavalia* spp. To date, cytogenetic studies on *C. gladiata* and *C. ensiformis* have been limited to karyomorphological descriptions of conventionally stained metaphase chromosome complements (Bhandari et al. 1969, Bairiganjan and Patnaik 1989, Li 1989, Rodrigues and Torne 1990, Chen 2003). The genome organization of the two species has not yet been determined using fluorochrome banding and fluorescence *in situ* hybridization (FISH).

Detailed karyotypes displaying chromosome morphology, heterochromatin distribution, and location of repetitive DNA sequences and bacterial artificial chromosome (BAC) have been constructed for many plant species. These are used to reveal chromosome-level genome organization, investigate the evolutionary relationships among related species, and integrate genetic and physical maps (Fuchs et al. 1998, Moscone et al. 1999, Hasterok et al. 2001, de Moraes et al. 2007, Hamon et al. 2009, Robledo et al. 2009, Fonsêca et al. 2010, Chacón et al. 2012, She et al. 2015, She and Jiang 2015, Zhang et al. 2015, Kirov et al. 2016). Karyotype analysis is often hampered by limitations in the ability to identify individual chromosomes due to a lack of markers. To overcome this obstacle, chromosome banding techniques such as Giemsa banding, fluorochrome banding, and FISH using repetitive DNA sequences and BAC clones as probes have been successively applied.

Combined propidium iodide (PI) and 4',6-diamidino-2-phenylindole (DAPI) staining (CPD staining; a type of fluorochrome banding) simultaneously reveals GC- and AT-rich chromosome regions (Peterson et al. 1999, Chaowen et al. 2004, She et al. 2006, She et al. 2015). The rRNA genes, 5S and 45S (18S-5.8S-26S) rDNA, have been widely applied in plants as repetitive DNA probes for FISH. The 45S rDNA is present in hundreds of repeated units arranged in tandem arrays. The 5S rDNA is also arranged in tandem arrays of hundreds to thousands of copies. Each 5S rDNA repeat unit consists of a coding region and a non-transcribed spacer (NTS). The coding region is approximately 120 bp and highly conserved across species. In contrast, the NTS regions show much intra- and inter-specific variability in length or nucleotide composition (Sastri et al. 1992). The NTS sequences of 5S rDNA have been used to study phylogenetic relationships among infrageneric taxa (Liu et al. 2003). The distribution patterns of rRNA genes revealed by FISH can be used as karyotype markers (Moscone et al. 1999, Hasterok et al. 2001, Chacón et al. 2012, She et al. 2015, She and Jiang 2015, Kirov et al. 2016). In a phylogenetic context, interpreting the changes in the number and location of rDNA loci in related species facilitates the understanding of the mechanisms and directions of chromosomal changes and their impact on plant evolution (e.g. Moscone et al. 2007, de Moraes et al. 2007, Chung et al. 2008, Weiss-Schneeweiss et al. 2008, Hamon et al. 2009, Robledo et al. 2009, Wolny and Hasterok 2009, She et al. 2015).

The GISH technique, a modification of FISH using genomic DNA as a probe, is conventionally utilized for identifying parental genomes in hybrids and allopolyploids (Schwarzacher et al. 1989). Two adaptations of the GISH technique, self-genomic *in situ* hybridization (sGISH) and comparative genomic *in situ* hybridization (cGISH), have been developed for plant genome analysis. In sGISH, the genomic DNA of a species is applied to its own chromosomes. It is an effective way to reveal the chromosomal distribution of repetitive DNA sequences in a given species (She et al. 2007, Falistocco and Marconi 2013, Zhang et al. 2015). In some plants, sGISH signal patterns permitted accurate identification of entire chromosomes or portions of them (She et al. 2007, Zhou et al. 2008, Zhang et al. 2015). In cGISH, the labeled total genomic DNA of one species is hybridized to the chromosomes of another species without the competitive DNA. It generates hybridization signals in the chromosomal regions of conserved repetitive DNA sequences. Therefore, it can be used to identify the phylogenetic relationships among related species (Falistocco et al. 2002, Wolny and Hasterok 2009, She et al. 2015, Zhang et al. 2015).

In the present study, molecular cytogenetic characterization of *C. gladiata* and *C. ensiformis* was performed using sequential CPD staining, dual color FISH with 5S and 45S rDNA probes, and sGISH. Detailed karyotypes of the two species were established using a combination of chromosome measurements, CPD bands, and rDNA-FISH and sGISH signals. cGISH of the genomic DNA of one species to the chromosomes of the other species was also performed. The 5S rDNA repeats of the two species were cloned, sequenced, and mapped using FISH. The data were assessed to gain insights into the evolutionary relationships between the two cultivated *Canavalia* species.

## Material and methods

### Plant materials and genomic DNA extraction

Seeds of *C. gladiata* (Jacq.) DC. were obtained from the Chinese Crop Germplasm Resources Information System (CGRIS) and collected in China. Seeds of *C. ensiformis* (L.) DC. were kindly provided by the United States (US) National Plant Germplasm System (NPGS) and collected in Brazil (PI 337078). For GISH and amplification of the 5S rDNA sequences, genomic DNA (gDNA) was extracted from young leaves using cetyltrimethylammonium bromide (CTAB) based on the method described by Murray and Thompson (1980).

### Amplification, cloning, and sequencing of 5S rDNA

The 5S rDNA sequences (including the coding regions and NTS) were amplified by polymerase chain reaction (PCR) using the specific primers 5S1 (5' -GGATGGGTGACCTCCCGGGAAGTCC-3') and 5S2 (5' -CGCTTAACTGCGGAGTTCTGATGGG-3') deduced from the 5S rRNA gene coding sequence of *Beta vulgaris* Linnaeus, 1753 (Schmidt et al. 1994). The PCR profile was as follows: denaturation at 94°C (3 min); 35 cycles at 94°C (1 min), 56°C (45 s), and 72°C (90 s); extension at 72°C for 10 min. The gel was purified using a PCR Product Purification Kit (Sangon Biotech, Shanghai, China). The PCR products were then ligated to pUCm-T vector using a Sangon Biotech PCR Cloning kit, transformed into *Escherichia coli* JM109 competent cells, and plated on selective medium with ampicillin. Clones were directly screened by PCR for the presence of inserts of the expected size. Five clones per species were amplified using the M13 forward and reverse primers then sequenced using the ABI PRISM 3730 DNA sequencer (Sangon Biotech). The DNA sequences of the five clones from each species were aligned to generate consensus sequences. Similarity searches were conducted on the BLAST site (http://blast.ncbi.nlm.nih.go) of the NCBI database. Using the ClustalW program in MEGA 4.0 (Tamura et al. 2007), the DNA sequences were aligned and the G + C content and variable sites were analyzed.

### Chromosome preparations

The procedure for mitotic chromosome preparation was essentially the same as that reported in published protocols (She et al. 2015). Seeds were germinated in the dark at 28°C on filter paper moistened with tap water. Actively growing root tips were pretreated with saturated α-bromonaphthalene for 1.0 h at 28°C then fixed in 3:1 (v/v) methanol/glacial acetic acid overnight. The root tips were then washed in double-distilled water and citrate buffer (0.01 mM citric acid-sodium citrate, pH 4.6) for 10 min each and incubated in a mixture of 1% cellulase RS (Yakult Pharmaceutical Industry, Tokyo, Japan), 1% pectolyase Y23 (Yakult Pharmaceuticals), and 1% cytohelicase (Sigma-Aldrich, Steinhem, Germany) in citric acid buffer at 28°C for 1.5 h. Root tips were transferred to a glass slide along with the fixative and dissected using fine-pointed forceps. Finally, the slides were dried above a flame and stored at −20°C.

### Staining with CPD

The CPD staining followed the procedure described in She et al. (2006). Chromosome preparations were treated with RNase A and pepsin then stained with a mixture of 0.6 µg ml^−1^ PI and 3 µg ml^−1^ DAPI (both from Sigma-Aldrich) in a 30% (v/v, using double-distilled water as solvent) solution of Vectashield H-1000 (Vector Laboratories Burlingame, USA). Preparations were examined under an Olympus BX60 epifluorescence microscope equipped with a CoolSNAP EZ CCD camera (Photometrics, Tucson, USA). The CCD camera was controlled using MetaMorph software (Molecular Devices, Sunnyvale, USA). Observations were made and photographs taken using a green excitation filter for PI and a UV excitation filter for DAPI. Greyscale images of each same plate were merged to produce a CPD image. The final images were optimized for contrast and background using PHOTOSHOP version 8.01 (Adobe).

### Probe DNA labeling

A 45S rDNA clone containing a 9.04-kb tomato 45S rDNA insert (Perry and Palukaitis 1990) and a pTa794 clone containing a 410-bp BamHI fragment of wheat 5S rDNA (Gerlach and Bedbrook 1979) were used as probes to localize the two ribosomal RNA gene families. They were labeled with biotin-16-dUTP and digoxigenin-11-dUTP, respectively, using Nick Translation Kit (Roche Diagnostics, Mannheim, Germany). The cloned 5S rDNA repeats and the gDNA from *C. gladiata* and *C. ensiformis* were labeled with digoxigenin-11-dUTP using the Nick Translation Kit. Approximately 1 μg plasmid or genomic DNA was used to label each probe.

### Fluorescence *in situ* hybridization

FISH with 5S and 45S rDNA probes and cGISH were carried out after CPD staining on the same slides. FISH with cloned 5S rDNA repeats and sGISH were conducted on the slides that were previously stained with CPD and hybridized with the 5S and 45S rDNA probes. The slides were then washed in 2× SSC (Saline-sodium citrate buffer) twice for 15 min each, dehydrated through an ethanol series (70%, 90%, and 100%, 5 min each), and used for hybridization. The *in situ* hybridization procedure followed the protocol described in detail by She et al. (2006). The biotin-labeled probe was detected using Fluorescein Avidin D (Vector Laboratories). The digoxigenin-labeled probe was detected by Anti-digoxigenin-rhodamine (Roche Diagnostics). Slides were counterstained and mounted with 3 µg ml^−1^ DAPI in 30% (v/v) Vectashield H-1000 and examined under an epifluorescence microscope fitted with a CCD camera. The chromosome spreads recorded in previous CPD and FISH experiments were examined. Grey-scale images were digitally captured using MetaMorph software with UV, blue and green excitation filters for DAPI, fluorescein, and rhodamine, respectively. The images were then merged and edited with PHOTOSHOP version 8.01 (Adobe).

### Karyotype analysis

For each species, five metaphase plates that had been subjected to sequential CPD staining, rDNA-FISH, and sGISH were measured using Adobe Photoshop version 8.01 to obtain chromosome relative lengths (RL; percentage of haploid complement), arm ratios (AR; long arm/short arm), fluorochrome band and sGISH signal sizes, and percent distance from the centromere to the rDNA site (di = *d* × 100/*a*; where *d* = distance from the middle of the rDNA sites to the centromere; *a* = corresponding chromosome arm length). The satellite length was included in the respective chromosome arm length. The stretched secondary constriction (SC) lengths were omitted. The total haploid complement length (TCL; the karyotype length) was measured using the five metaphase cells with the highest degree of chromosome condensation. The arm ratios were used to classify the chromosomes according to the system described by Levan et al. (1964). Chromosomes were identified and idiograms were drawn based on the measurements, fluorochrome bands, rDNA-FISH signals, and sGISH signals. The chromosomes in the karyotype were arranged by order of decreasing size. Karyotype asymmetry was determined using the mean centromeric index (CI), the intrachromosomal asymmetry index (A1), the interchromosomal asymmetry index (A2) (Romero Zarco 1986), the ratio of long arm length in chromosome set to total chromosome length in set (As K%) (Arano 1963), the asymmetry index (AI) (Paszko 2006), and the categories of Stebbins (1971).

## Results

### Characterization of 5S rDNA repeats

For both species, genomic DNA amplification produced one major fragment of approximately 950 bp and one minor fragment of approximately 450 bp. Amplicons were cloned. Ten from each transformation were screened to verify the presence of the insert. Five clones of each fragment were sequenced.

**Figure 1.**
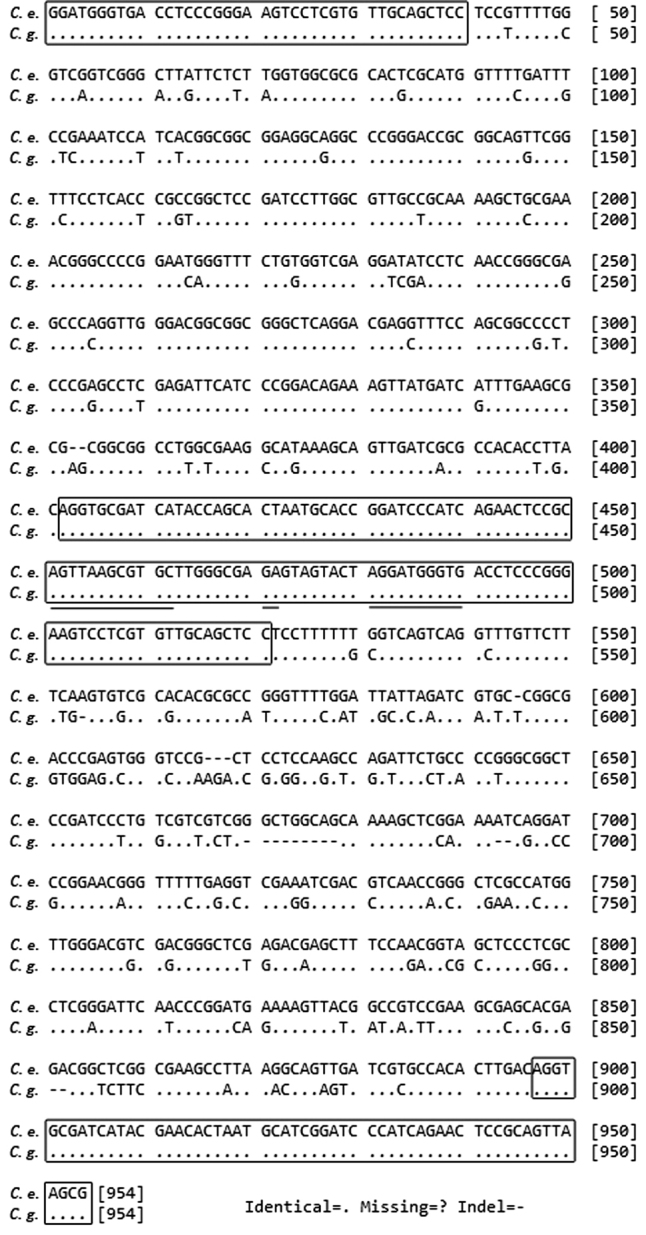
Alignment of the major fragments amplified from the 5S rDNA repeats of *Canavalia gladiata* (C. g.) and *Canavalia ensiformis* (C. e.). The entire 120-bp 5S rRNA gene and the 40 and 58 bp of the gene flanking the 5' and 3' ends are enclosed in a box; the intragenic promoter motifs are underlined.

Sequence analysis showed that all inserts correspond to 5S rDNA repeats. Each fragment was neighbored by 40 bp and 58 bp of the gene at the 5' and the 3' ends, respectively (Fig. 1). There was complete homology among the transcribed regions of the fragments. The minor fragments (459 bp and 457 bp amplified from *C. gladiata* and *C. ensiformis*, respectively) included the entire 361 bp NTS (in *C. gladiata*) or 359 bp NTS (in *C. ensiformis*). The major fragments (940 bp and 948 bp amplified from *C. gladiata* and *C. ensiformis*, respectively) consisted of two NTS regions separated by the whole gene sequence. The major fragments were deposited in the GenBank database (accession numbers: KU230029.1 and KU230030.1). The 5' and 3' end NTS regions of the major fragment from *C. gladiata* were both 361 bp but differed in nucleotide composition (variable sites: 35/361; G + C contents: 62.9% and 60.3%, respectively). The 5' and 3' end NTS regions of the major fragment from *C. ensiformis* differed in length (359 bp and 371 bp, respectively) and in nucleotide composition (variable sites: 100/375; G + C contents: 62.4% and 59.1%, respectively). There was a lower level of sequence identity (variable sites: 145/736; identity value: 80.3%) between *C. ensiformis* and *C. gladiata* in terms of the 5' and 3' end NTS regions of their major fragments. The 5S rRNA genes consist of a conserved 120-bp sequence starting with AGG and ending with TCC. According to the BLAST site of the NCBI database, this configuration is almost identical to those of *Vigna angularis* (Willdenow, 1800) Ohwi & H.Ohashi, 1969, *Vigna radiata* (Linnaeus, 1753) R. Wilczek, 1954, *Lupinus luteus* Linnaeus, 1753, *Glycine max* (Linnaeus, 1753) Merrill, 1917 and other Fabaceae species (Gottlob-McHugh et al. 1990, Nuc et al. 1993, Sakai et al. 2015). An intragenic promoter composed of an A-box, an Intermediate Element (IE), and a C-box was identified (Fig. 1) by comparing the 5S rDNA gene sequences of the two *Canavalia* species with that of *Arabidopsis thaliana* (Linnaeus, 1753) Heynhold, 1842 (Cloix et al. 2003).

### General karyotype features

**Figure 2.**
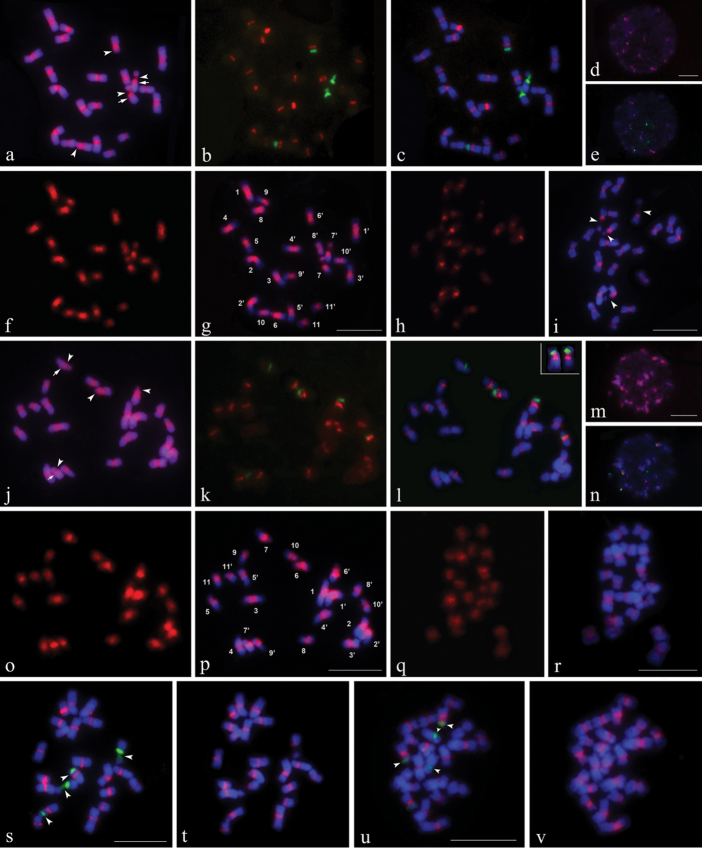
Mitotic chromosomes (except for **d, e, m, n**) and interphase nuclei (**d, e, m** and **n**) of *Canavalia gladiata* (**a–i, s, t**) and *Canavalia ensiformis* (**j–r, u, v**) after sequential CPD staining and *in situ* hybridization. **a, d, j, m** CPD-stained chromosomes and interphase nuclei. **c, e, l, n, s, u** Chromosomes and interphase nuclei showing 5S (red) and 45S (green) rDNA signals produced by digoxigenin-labeled 5S rDNA and biotin-labeled 45S rDNA probes. **b** and **k** 5S and 45S rDNA signals only. **f** and **o** Signals produced by digoxigenin-labeled total genomic DNA of their own, **g** and **p** Chromosomes with sGISH signals. **h** and **q** Signals produced by digoxigenin-labeled total genomic DNA probes from other species. **i** and **r** Chromosomes with cGISH signals. **t** and **v** FISH of digoxigenin-labeled 5S rDNA repeats cloned from *C. gladiata* and *C. ensiformis* to same spreads shown in **s** and **u**, respectively. Arrows in **a** and **j** indicate positions of pair 7 centromeres. Arrowheads in **a, i, j, s** and **u** indicate distinguishable secondary constrictions (SC). Chromosome numbers in **g** and **p** are designated by karyotyping. Chromosomes in upper right corner of **l** are pair 6 from another spread showing proximal 5S rDNA loci on short arms. Chromosomes were counterstained using DAPI (blue). Bars = 10 µm.

**Figure 3.**
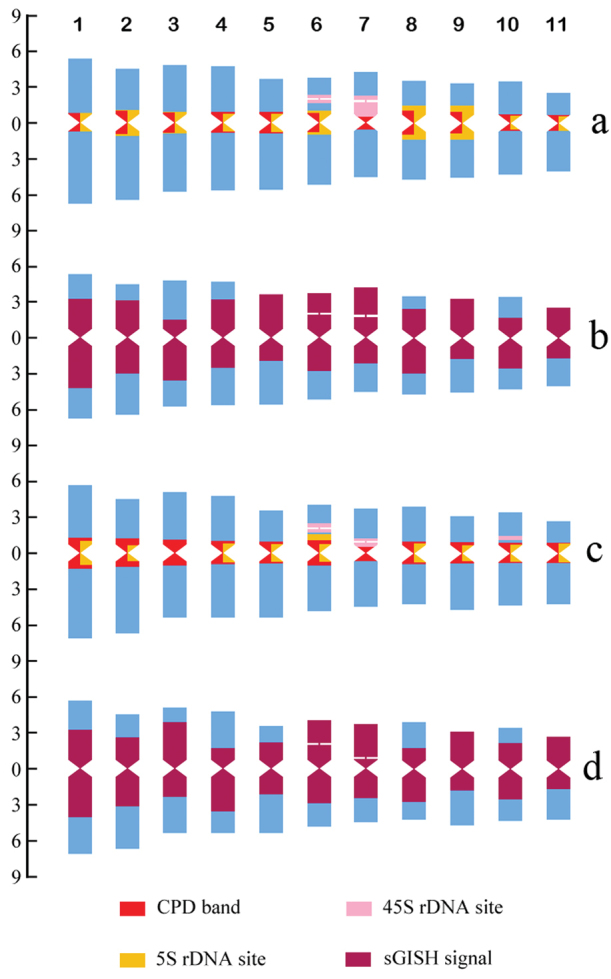
Idiograms of *Canavalia gladiata* (**a, b**) and *C. ensiformis* (**c, d**). **a** and **c** are idiograms displaying chromosome measurements and position and size of fluorochrome bands and rDNA FISH signals, **b** and **d** are idiograms displaying chromosome measurements and size and distribution of sGISH signals. Ordinate scale on left indicates relative chromosome length (% of haploid complement). The numbers above panel **a** are chromosome numbers.

Representative mitotic chromosomes of C. *gladiata* and *C. ensiformis* are shown in Figure 2. The chromosome measurements for both species are given in Table 1. Idiograms displaying the chromosome measurements, position and size of the CPD bands, rDNA-FISH signals, and sGISH signals are illustrated in Figure 3.

**Table 1.** Chromosome measurements of *Canavalia gladiata* (*C.g.*) and *Canavalia ensiformis* (*C.e.*) obtained from five metaphases per species.

**Species**	**Chr. No.**	**Relative length (%)**			**Arm ratio ± SD**	**Type**	**Centromeric CPD band size^‡^ ± SD**	**sGISH signal size^‡^**		
		**Short arm ± SD**	**Long arm ± SD**	**Total ± SD**				**Short arm ± SD**	**Long arm ± SD**	**Total ± SD**
*C. g.*	1	5.25 ± 0.23	6.70 ± 0.24	11.95 ± 0.41	1.28 ± 0.05	m	1.53 ± 0.27	3.19 ± 0.19	4.17 ± 0.13	7.35 ± 0.22
	2	4.43 ± 0.10	6.39 ± 0.36	10.82 ± 0.31	1.44 ± 0.10	m	1.93 ± 0.15	3.07 ± 0.33	2.96 ± 0.19	6.03 ± 0.38
	3	4.72 ± 0.37	5.69 ± 0.19	10.40 ± 0.28	1.21 ± 0.12	m	1.63 ± 0.17	1.44 ± 0.28	3.56 ± 0.13	5.00 ± 0.28
	4	4.63 ± 0.20	5.57 ± 0.17	10.20 ± 0.17	1.21 ± 0.08	m	1.67 ± 0.14	3.19 ± 0.22	2.50 ± 0.15	5.69 ± 0.09
	5	3.61 ± 0.08	5.53 ± 0.09	9.15 ± 0.16	1.53 ± 0.02	m	1.76 ± 0.19	3.61 ± 0.08	1.94 ± 0.24	5.55 ± 0.31
	6	3.71 ± 0.46	5.11 ± 0.22	8.83 ± 0.61	1.39 ± 0.16	m†	1.54 ± 0.10	3.71 ± 0.46	2.79 ± 0.26	6.50 ± 0.67
	7	4.14 ± 0.20	4.50 ± 0.26	8.64 ± 0.38	1.09 ± 0.06	m^†^	1.14 ±0.18	4.14 ± 0.20	2.13 ± 0.32	6.27 ± 0.50
	8	3.42 ± 0.25	4.68 ± 0.12	8.11 ± 0.21	1.38 ± 0.13	m	1.93 ± 0.27	2.33 ± 0.23	3.01 ± 0.14	5.34 ± 0.31
	9	3.20 ± 0.08	4.53 ± 0.20	7.73 ± 0.18	1.42 ± 0.08	m	1.79 ± 0.11	3.20 ± 0.08	1.77 ± 0.21	4.97 ± 0.25
	10	3.39 ± 0.10	4.31 ± 0.29	7.70 ± 0.38	1.27 ± 0.06	m	1.36 ± 0.16	1.62 ± 0.28	2.55 ± 0.32	4.18 ± 0.31
	11	2.44 ± 0.16	4.03 ± 0.21	6.47 ± 0.27	1.65 ± 0.13	m	1.32 ± 0.12	2.43 ± 0.18	1.73 ± 0.17	4.15 ± 0.33
	Total	42.96 ± 0.51	57.04 ± 0.51	100			17.59 ± 1.13	31.93 ± 0.33	29.11 ± 0.32	61.04 ± 0.19
*C. e.*	1	5.58 ± 0.23	7.02 ± 0.38	12.60 ± 0.18	1.26 ± 0.11	m	2.54 ±0.57	3.18 ± 0.17	3.99 ±0.29	7.17 ±0.36
	2	4.43 ± 0.18	6.62 ± 0.28	11.05 ± 0.38	1.50 ± 0.07	m	2.32 ±0.63	2.53 ± 0.29	3.14 ±0.20	5.66 ±0.40
	3	5.02 ± 0.16	5.33 ± 0.60	10.35 ± 0.51	1.06 ± 0.14	m	2.14 ±0.68	3.77 ± 0.24	2.32 ±0.11	6.10 ±0.29
	4	4.68 ± 0.16	5.35 ± 0.43	10.03 ± 0.53	1.14 ± 0.08	m	1.93 ±0.40	1.67 ± 0.21	3.57 ±0.19	5.24 ±0.37
	*5*	3.49 ± 0.19	5.36 ± 0.28	8.85 ± 0.29	1.54 ± 0.13	m	1.80 ±0.23	2.12 ± 0.17	2.13 ±0.32	4.25 ±0.31
	*6*	3.96 ± 0.29	4.80 ± 0.25	8.76 ± 0.46	1.22 ± 0.08	m^†^	2.08 ±0.51	3.96 ± 0.29	2.87 ±0.24	6.83 ±0.31
	*7*	3.65 ± 0.25	4.46 ± 0.16	8.11 ± 0.15	1.23 ± 0.13	m^†^	1.45±0.18	3.65 ± 0.25	2.48 ±0.55	6.13 ±0.57
	*8*	3.76 ± 0.25	4.25 ± 0.28	8.01 ± 0.41	1.13 ± 0.10	m	1.88 ±0.33	1.65 ± 0.22	2.73 ±0.15	4.38 ±0.34
	*9*	2.99 ± 0.23	4.73 ± 0.32	7.72 ± 0.23	1.59 ± 0.22	m	1.76 ±0.20	2.99 ± 0.23	1.81 ±0.19	4.80 ±0.35
	10	3.35 ± 0.24	4.34 ± 0.22	7.69 ± 0.44	1.30 ± 0.05	m	1.67 ±0.18	2.06 ± 0.15	2.56 ±0.26	4.62 ±0.37
	11	2.59 ± 0.27	4.23 ± 0.19	6.82 ± 0.44	1.64 ±0.12	m	1.68 ±0.08	2.59 ± 0.27	1.76 ±0.38	4.35 ±0.61
	Total	43.50 ± 0.76	56.50 ± 0.76	100			21.24 ± 3.11	30.18 ± 1.36	29.35 ± 2.27	59.53 ± 3.50

SD, standard deviation. m, metacentric. ^†^satellite chromosome (satellite length was included in chromosome length but secondary constriction length was excluded). ^‡^ % of band (signal) size in relation to the karyotype length.

Both *C. gladiata* and *C. ensiformis* have a diploid chromosome number 2n = 22. The mitotic metaphase chromosomes are rather small. The TCL for *C. gladiata* and *C. ensiformis* are 40.46 ± 1.03 μm and 34.06 ± 3.87 μm, respectively. The individual metaphase chromosomes ranged from 4.72-2.63 μm long in *C. gladiata*, and from 4.21-2.43 μm long in *C. ensiformis*.

Both species have karyotypes composed of metacentric (m) chromosomes only (Table 1; Fig. 3). Chromosome pairs 6 and 7 have satellites with secondary constrictions (SC) located at the interstitial and proximal positions of the short arms, respectively (Fig. 2a, c, i, s, j, l, u). The karyotypes were therefore formulated as 2n = 22 = 18m + 4m-SAT. In most prometaphase (images not shown) and some metaphase cells, the satellites were visualized separately from the rest of the chromosomes with the SC stretched (Fig. 2a, i). At metaphase, the SC of pair 7 in *C. gladiata* stretched more frequently than did that in *C. ensiformis*. Six asymmetry indices, CI, A1, A2, As K%, AI, and the Stebbins’ category, are 42.78±2.92, 0.25, 0.18, 57.04, 1.23, and 1A for *C. gladiata*, and 43.31±3.66, 0.23, 0.19, 56.50, 1.63, and 1A for *C. ensiformis*. These data indicate that both karyotypes are similar and symmetric; however, based on AI, the karyotype of *C. ensiformis* is slightly asymmetrical relative to that of *C. gladiata*.

### CPD banding patterns

CPD staining revealed that both species had similar fluorochrome banding patterns. The centromeric regions of all chromosome pairs and the 45S rDNA sites demonstrated by sequential rDNA-FISH appeared as red CPD bands (Fig. 2a, j). The pair 6 rDNA CPD bands did not significantly differ in size and intensity between the two species. Nevertheless, the pair 7 rDNA CPD bands of *C. gladiata* were larger and more intense than those of *C. ensiformis*. The pair 10 rDNA CPD bands of *C. ensiformis* were juxtaposed with the centromeric CPD bands. The primary constrictions of pair 7 in both species were not as obvious as those of other pairs and were assumed to be adjacent to the proximal regions of the rDNA CPD bands. They displayed small, weak CPD bands (Fig. 2a, j). The size of the centromeric CPD bands was expressed as a percentage of the karyotype length and ranged from 1.14–1.93% in *C. gladiata*, and 1.45-2.54% in *C. ensiformis*. The centromeric bands of *C. gladiata* occupied 17.59% and those of *C. ensiformis* took up 21.24% of the total karyotype length (Table 1; Fig. 3). Up to 24 red-fluorescing heterochromatin blocks of different sizes were observed in the CPD-stained interphase nuclei of both species (Fig. 2d, m).

### rDNA FISH patterns

FISH analyses of the 5S and 45S rDNA probes to the CPD-stained mitotic chromosomes and interphase nuclei are presented in Fig. 2. Ten 5S rDNA loci were detected in both species. In *C. gladiata*, the 5S signals were observed in the centromeres of all but the seventh chromosome pair and were strongest for pair 9 and weakest for pair 10 (Fig. 2b, c, s). In *C. ensiformis*, 5S signals were found in the centromeres of all but the 3^rd^ and 7^th^ pairs, and the proximal regions of the short arms of pair 6 (*di* = 32.07%). There were no significant differences in intensity (Fig. 2k, l, u). In interphase cells, the 5S signals were all co-localized with the CPD-banded heterochromatin blocks (Fig. 2e, n). Two and three loci for 45S rDNA were detected in *C. gladiata* and *C. ensiformis*, respectively. Two pairs of 45S signals associated with the SC of the satellite chromosome pairs 6 and 7 were detected in both species (*di* = 54.32% for pair 6 and 38.67% for pair 7 in *C. gladiata*; *di* = 50.57% for pair 6 and 26.14% for pair 7 in *C. ensiformis*). These correspond to their respective CPD bands in both size and intensity (Fig. 2a, b, c, j, k, l, s, u). In *C. ensiformis*, a minor 45S locus was observed in the proximal regions of the short arms of pair 10 (*di* = 29.05%; Fig. 2k, l, u). The 45S signals of pair 6 for both *C. gladiata* and *C. ensiformis* were similar in intensity. The 45S signals of pair 7 in *C. gladiata* were much stronger than those in *C. ensiformis* (Fig. 2b, c, k, l, s, u). At interphase, dispersed 45S signals were found. These consisted of four or six strongly fluorescing knobs with varying numbers of weakly fluorescing spots emanating from them (Fig. 2e, n).

FISH performed on mitotic chromosomes using the cloned major 5S rDNA fragment probe generated signals in the regions corresponding to the 5S signals from pTa794 and in the centromeres wherein no signal was generated using pTa794 (Fig. 2t, v). The signals from the cloned major 5S rDNA fragments were slightly larger and stronger than those produced by pTa794 (Fig. 2s, t, u, v).

### Self-GISH signal patterns

The chromosomal distribution patterns of repetitive DNA sequences were investigated using self-GISH. Distinct sGISH signal patterns were generated in both species and they were largely similar to each other (Figs 2f, g, o, p; 3b, d). sGISH signals appeared on each chromosome in the complement and accounted for 61.04% of the total karyotype length in *C. gladiata* and 59.53% in *C. ensiformis* (Table 1). The size of the sGISH signal in each chromosome pair was expressed as a percentage of the karyotype length. It varied from 4.15–7.35% in *C. gladiata* and from 4.25–7.17% in *C. ensiformis* (Table 1; Fig. 3b, d). The signals were distributed in all pericentromeric regions, the proximal regions of some chromosome arms, and entire short arms of certain chromosome pairs. The genomic probe intensely labeled the 45S rDNA sites in both species. The distal regions of most chromosome arms (17–18 arms of the haploid complement) had no fluorescence. In particular, the size and location of the sGISH signal of each chromosome pair are unique and, along with the measurements and rDNA-FISH signals, enable each metaphase chromosome to be identified accurately (Figs 2g, p; 3b, d). In *C. gladiata*, the short arms of pairs 5, 6, 7, 9, and 11 were entirely labeled. The signal sizes on both the short and long arms of pairs 1, 2, 6, 8, and 11 were similar. The signal sizes on the long arms of pairs 3 and 10 were much larger than those on their short arms. The signal sizes on the long arms of pairs 4, 5, 7, and 9 were much lower than those on their short arms (Figs 2g; 3b). In *C. ensiformis*, the signal patterns of pairs 1, 2, 6, 7, 9, and 11 resembled the same ones in *C. gladiata*. The signal patterns of pairs 3 and 4 in *C. ensiformis* resembled those of pairs 4 and 3 of *C. gladiata*, respectively. The short arm of pair 5 was not entirely labeled. The distal regions lacked any fluorescent signal. In contrast, for *C. ensiformis*, the signal of the short arm of pair 8 decreased and that of pair 10 increased relative to those in *C. gladiata* (Figs 2p; 3d). For both species, the total amounts of sGISH signal in both short and long arms of the complement were nearly the same (Table 1).

### Comparative GISH signal patterns

cGISH was employed to probe the gDNA signals on the metaphase chromosomes of another species (Fig. 2h, i, q, r) to reveal the homology of repetitive DNA sequences between the two species. On the metaphase chromosomes of *C. gladiata*, the gDNA of *C. ensiformis* generated signals in all pericentromeric regions and 45S rDNA sites. Most centromeres and the 45S rDNA sites of pair 7 were strongly labeled compared with other regions (Fig. 2h, i). In *C. ensiformis*, cGISH with *C. gladiata* gDNA also produced strong signals in all pericentromeric regions and 45S rDNA sites. The highest intensity was observed at the centromeres (Fig. 2q, r).

## Discussion

### Characteristics of the two *Canavalia* genomes

In this study, detailed karyotypes of *C. gladiata* and *C. ensiformis* were established using a combination of chromosome measurements, CPD bands, rDNA-FISH signals, and sGISH signals. The karyotypes provided the first molecular cytogenetic characterization of the two cultivated *Canavalia* species. The sGISH and rDNA-FISH signals were effective cytogenetic markers enabling unambiguous identificaion of individual chromosomes in both species.

The data revealed that the karyotypes of both *C. gladiata* and *C. ensiformis* are quite symmetrical. The karyotype of *C. ensiformis* has not been reported previously. The karyotype of *C. gladiata* in the present study shows more symmetry and differs from those described by Li (1989), Bairiganjan and Patnaik (1989), and Chen (2003). Discrepancies in karyotype formula were probably due to differences in the material analyzed and difficulties in identifying chromosomes using classical staining techniques.

The rDNA-FISH revealed that there are a substantial number of 5S rDNA loci located in the centromeres in both species. There should be 5S rDNA repeats in all centromeres in both species because FISH using the cloned major 5S rDNA fragment generated weak signals in the centromeres wherein no signal was detected by pTa794. The copy number of 5S rDNA repeats within the centromeres of pair 7 (both species) and pair 3 of *C. ensiformis* was probably too low to be detected by FISH using the exogenous 5S rDNA probe. Centromeric 5S rDNA arrays have seldom been detected in plants by FISH. One to several centromeric 5S loci have only been reported for two *Grindelia* (Willdenow, 1807) species (Baeza and Schrader 2005), *Podophyllum hexandrum* Royle, 1834 (Nag and Rajkumar 2011), *Paphiopedilum* Pfitzer, 1886 (Lan and Albert 2011), two *Alstroemeria* (Linnaeus, 1762) species (Chacón et al. 2012), and *Vigna aconitifolia* (Jacquin, 1768) Maréchal, 1969 (She et al. 2015). The centromeric regions in plants, including Phaseoleae species, consist of different satellite DNA families and transposable elements (Jiang et al. 2003, Tek et al. 2010, Iwata et al. 2013). The 5S rDNA signals may not actually be located in the functional regions of the centromeres even though they seemed to coincide exactly with them. It is worth verifying whether 5S rDNA repeats participate in centromere function using immunofluorescence and chromatin immunoprecipitation (ChIP)-based assays (Tek et al. 2010, Iwata et al. 2013).

Another prominent feature of the two *Canavalia* genomes was the non-rDNA GC-rich heterochromatin in all centromeres (highlighted by CPD staining) (She et al. 2006). Centromeric, pericentromeric, or proximal non-rDNA GC-rich heterochromatin have been observed in many Phaseoleae, including *Psophocarpus tetragonolobus* A. P. de Candolle, 1825 (Chaowen et al. 2004), four cultivated *Phaseolus* (Linnaeus, 1754) species (Bonifácio et al. 2012), seven cultivated *Vigna* (Savi, 1824) species (She et al. 2015), Lablab purpureus (Linnaeus, 1763) Sweet, 1826 (She and Jiang 2015), and *Crotalaria* (Linnaeus, 1753) species from two sections of the tribe Crotalarieae (Mondin and Aguiar-Perecin 2011) which is a branch of the Genistoid clade (LPWG 2013). A recent multilocus phylogenetic analysis reestablished the tribe Diocleae as a branch of the Phaseoloid (Millettioid) clade, which includes the *Canavalia* and two other clades (Queiroz et al. 2015). It is therefore proposed that the presence of (peri)centromeric GC-rich heterochromatin is an ancestral characteristic existing before the origin of Phaseoloid (LPWG 2013). In the two *Canavalia* species, however, most centromeric CPD bands should arise when 5S rDNA repeats intersperse with other GC-rich repeats. All but one centromeric CPD band in *C. gladiata* and two in *C. ensiformis* were co-localized with 5S rDNA arrays. Nevertheless, they did not completely correspond in size to the 5S signals. The sequence analysis revealed the NTS of 5S rDNA repeats of both species was GC-rich. GC-rich regions co-localized with 5S rDNA sites have also been observed in other plants (e.g. Zoldos et al. 1999, Hamon et al. 2009, She et al. 2015).

The sGISH experiments revealed a distinct distribution of repetitive DNA sequences on the chromosomes of the two *Canavalia* species. sGISH data obtained from many plants showed that the chromosomal distribution of repetitive sequences is often non-uniform and forms clusters within heterochromatin blocks, and two different sGISH patterns may occur depending on the genome size of the species (She et al. 2007). In plants with small, compact genomes, the hybridization signals concentrate mainly in (peri)centromeric or proximal regions, heterochromatic arms, and 45S rDNA sites (Falistocco et al. 2002, Maluszynska and Hasterok 2005, She et al. 2007, Wolny and Hasterok 2009, Falistocco and Marconi 2013, She et al. 2015). In plants with large genomes, the entire chromosome length is densely labeled with strongly and weakly labeled regions alternate, or with enhanced signals located in C-band regions (She et al. 2007, Zhou et al. 2008). The repetitive sequence distribution patterns in *C. gladiata* and *C. ensiformis* generally resemble those of small plant genomes reported previously but had their own unique characteristics not reported elsewhere. The repetitive sequences are distributed asymmetrically on both sides of the centromeres, unequally between chromosome pairs, but evenly between the short and long arms in the complement.

### Similarities and differentiation between the two *Canavalia* genomes

The molecular cytogenetic data obtained in this study revealed a high degree of similarity in genome organization between the two *Canavalia* species. This result confirms the evolutionary closeness between *C. gladiata* and *C. ensiformis* which was previously inferred from morphological and seed protein comparisons (Smartt 1990) and molecular phylogenetic analysis (Snak et al. 2016). Both species had the same karyotype formula and similar karyotype indices. The chromosome arrangements in the complement did not differ except for the exchange of pairs 3 and 4. The distributions of their centromeric CPD bands were similar. Most of their chromosome pairs had similar sGISH signal patterns. The 45S loci on pairs 6 and 7 and the centromeric 5S rDNA loci of nine chromosome pairs were conserved. The seventh chromosome pair lacked 5S rDNA signals. Extensive cross-hybridization and highly similar signal patterns resulted from reciprocal cGISH, which indicates high repetitive DNA homology and reflects their close phylogenetic relationships (Maluszynska and Hasterok 2005, She et al. 2015, Zhang et al. 2015).

The data also revealed distinct differences between the two genomes. The genome size of *C. ensiformis* was nearly one-sixth less than that of *C. gladiata* based on their TCL (Levin 2002). Rodrigues and Torne (1990) reported that the TCL of *C. ensiformis* was only 70.55% that of *C. gladiata*. The karyotype of *C. ensiformis* was more asymmetrical than that of *C. gladiata*. *C. ensiformis* with its annual life form and a more restained and bushier growth habit is considered to be more advanced in evolution than *C. gladiata*, which is closer to the wild species with its perennial life form and twining growth habit (Smartt 1990). Our karyotypic data support this opinion since a symmetrical karyotype is considered characteristic of more primitive species (Stebbins 1971). Furthermore, the karyotypic differences between the two species coincide with a karyotype evolutionary pattern in which increasing specialization is accompanied by genome size reduction, particularly where the specialization involves a shift to an annual habit or a shorter growing season. This downsizing results in an increase in karyotype asymmetry (Levin 2002). Nevertheless, detailed karyotyping revealed that the significant genome size contraction in *C. ensiformis* did not significantly change its karyotype morphology and complement sGISH signal proportion and distribution relative to those of *C. gladiata*. Therefore, the karyotypic comparison between the two species corroborates the increasing karyotype asymmetry hypothesis proposed by Levin (2002). This theory proposed that genome contraction is achieved by an equal reduction in the amount of DNA per chromosome regardless of chromosome size. This mechanism increases asymmetry.

Compared to *C. gladiata*, *C. ensiformis* gained an extra proximal 45S rDNA locus and a non-centromeric 5S rDNA locus but lost a centromeric 5S rDNA locus. Based on the signal intensity (Maluszynska and Heslop-Harrison 1991), the number of 45S rDNA repeats in pair 7 and 5S rDNA repeats in pairs 9 and 10 of *C. ensiformis* changed significantly. Differentiation among species in the chromosomal organization of rDNA clusters has been found in many genera and correlates with chromosome evolution during speciation (e.g. Moscone et al. 1999, 2007, Datson and Murray 2006, Chung et al. 2008, Weiss-Schneeweiss et al. 2008, Morales et al. 2012, She et al. 2015). As mentioned above, *C. gladiata* is closer to wild species than is *C. ensiformis*. Therefore, the rDNA pattern of *C. ensiformis* may have evolved from that of *C. gladiata*. The proximal 45S rDNA locus might have originated from the transposition of the SC-associated 45S rDNA cluster (Datson and Murray 2006, Chung et al. 2008). The proximal 5S locus on the short arms of pair 6 may have arisen from an inversion of the segment bearing part of the centromeric 5S rDNA (Moscone et al. 2007, Weiss-Schneeweiss et al. 2008). The disappearance of the 5S rDNA signal at the centromeres of pair 3 may have come from the significant reduction of 5S rDNA repeats in this region (Chung et al. 2008).

sGISH revealed that the distribution of repetitive sequences on pairs 5, 8, and 10, differed significantly between the two species. This fact suggests that *C. ensiformis* lost repetitive DNAs in some chromosomal regions and/or its chromosomes were rearranged during its evolution. Sequence analysis of 5S rDNA repeats revealed a lower level of NTS sequence identity between the species, indicating that their genomic sequences were clearly differentiated (Liu et al. 2003). The percentage of centromeric CPD bands in the complement of *C. ensiformis* was one-fifth (20%) greater than that of *C. gladiata*, reflecting an increase of the proportion of GC-rich heterochromatin in *C. ensiformis* (She et al. 2006).

## Conclusions

Individual chromosomes of both *C. gladiata* and *C. ensiformis* can be accurately identified by sGISH and rDNA-FISH signals.

Both *C. gladiata* and *C. ensiformis* genomes have particular characteristics including existence of non-rDNA GC-rich heterochromatin at all centromeres and 5S rDNA loci at the vast majority of centromeres, and a unique chromosomal distribution of repetitive DNA sequences.

Molecular cytogenetic comparison revealed both basic similarities and distinct differences in genome organization between *C. gladiata* and *C. ensiformis*, providing insights into the evolutionary relationships between them.
